# Associations of ECP (eosinophil cationic protein)-gene polymorphisms to allergy, asthma, smoke habits and lung function in two Estonian and Swedish sub cohorts of the ECRHS II study

**DOI:** 10.1186/1471-2466-10-36

**Published:** 2010-06-09

**Authors:** Ulla-Britt Jönsson, Lena Douhan Håkansson, Rain Jõgi, Christer Janson, Per Venge

**Affiliations:** 1 Department of Medical Sciences, Clinical Chemistry, University of Uppsala, Uppsala, Sweden; 2 Lung Clinic of Tartu University Hospital, Tartu, Estonia; 3 Respiratory Medicine, University of Uppsala, Uppsala, Sweden

## Abstract

**Background:**

The Eosinophil Cationic Protein (ECP) is a potent multifunctional protein. Three common polymorphisms are present in the ECP gene, which determine the function and production of the protein. The aim was to study the relationship of these ECP gene polymorphisms to signs and symptoms of allergy and asthma in a community based cohort (The European Community Respiratory Health Survey (ECRHS)).

**Methods:**

Swedish and Estonian subjects (n = 757) were selected from the larger cohort of the ECRHS II study cohort. The prevalence of the gene polymorphisms ECP434(G>C) (rs2073342), ECP562(G>C) (rs2233860) and ECP c.-38(A>C) (rs2233859) were analysed by DNA sequencing and/or real-time PCR and related to questionnaire-based information of allergy, asthma, smoking habits and to lung functions.

**Results:**

Genotype prevalence showed both ethnic and gender differences. Close associations were found between the ECP434(G>C) and ECP562(G>C) genotypes and smoking habits, lung function and expression of allergic symptoms. Non-allergic asthma was associated with an increased prevalence of the ECP434GG genotype. The ECP c.-38(A>C) genotypes were independently associated to the subject being atopic.

**Conclusion:**

Our results show associations of symptoms of allergy and asthma to ECP-genotypes, but also to smoking habits. ECP may be involved in impairment of lung functions in disease. Gender, ethnicity and smoking habits are major confounders in the evaluations of genetic associations to allergy and asthma.

## Background

The Eosinophil Cationic Protein (ECP), also named RNase 3, is a multifunctional protein with both cytotoxic and fibrogenic properties [1-3]. High levels of ECP were found in blood, bronchoalveolar lavage, saliva and sputum of subjects with allergic asthma indicating the presence of activated eosinophils in the disease process [1,4]. Recently several common polymorphisms were described in the ECP gene [5]. One of these polymorphisms ECP434(G>C) affects the protein coding part of ECP and gives rise to a change in the amino acid arginine at position 97 to threonine [2]. This substitution dramatically alters the function of ECP from a cytotoxic to a non-cytotoxic molecule. Another polymorphism was found in the 3'UTR region ECP562(G>C) and shown to be related to the cellular content of ECP [6]. A third polymorphism was seen in the intron of the ECP gene, ECP c.-38(A>C), and shown by others to be related to serum levels of ECP [7]. We found previously a close relationship between the ECP434(G>C) polymorphism and the expression of allergic symptoms such as asthma [8] whereas another group showed associations between the ECP c.-38(A>C) polymorphism and various symptoms of allergy and asthma and a relation to non-allergic asthma of the ECP434(G>C) polymorphism [7]. In an unpublished pilot study we found lower lung function, as measured with FEV_1_, in smokers who carried the ECP434GG genotype.

The aim of this study was to further investigate the possible relationships between the three common ECP gene polymorphisms to allergy/asthma and lung function. For this purpose we have genotyped a sub cohort of the European Community Respiratory Health Survey II (ECRHS II) study cohort [9] and related the genotypes to questionnaire-based information of allergy, asthma and smoke habits as well as to various measures of lung function.

## Methods

### Subjects

The European Community Respiratory Health Survey (ECRHS) is an international multi-centre study of asthma and allergy. The first part, ECRHS I, was conducted in 1990-1994 and the follow-up study, ECRHS II, in 1999-2001. The design of ECRHS I and ECRHS II has been published in detail [9,10]. Each participant was sent a brief questionnaire (Stage 1) and from those who responded, a random sample was invited to undergo a more detailed clinical examination (Stage 2). In addition a "symptomatic sample" consisting of those who reported symptoms of wakening with shortness of breath, asthma attacks or using asthma medication in stage 1 was also studied. In ECRHS II subjects who had participated in stage 2 of ECRHS I were invited to participate in a follow-up study. Subjects answered a standardised questionnaire administered by trained interviewers and underwent lung function test and blood test. Stage 2 of ECRHS I included 1382 subjects from Uppsala in Sweden and Tartu in Estonia. Of these, 1054 subjects were from the random sample and 328 from the symptomatic sample. The ECRHS II in Uppsala and Tartu included 1007 subjects of which DNA was available from 757 subjects. Of these 757 subjects 574 were from the randomly selected sample and 183 from the 'symptomatic sample'. The demographics of the randomly selected cohort is shown in Table 1 and further described below. The study was approved by the local ethics committees of Tartu, Estonia and Uppsala, Sweden. All subjects included in the study gave their informed consent to participate.

**Table 1:** Differences and similarities in the randomly selected cohorts of Sweden and Estonia

	Sweden	Estonia	All	p-value (χ^2^-test)
Men/Women	185/169 (52%/48%)	96/130 (42%/58%)	281/299 (48%/52%)	0.021
Never-smokers/Ex-smokers/Smokers	171/124/53 (49%/36%/15%)	107/30/89 (47%/13%/39%)	278/154/142 (48%/27%/25%)	< 0.0001
Atopy, no/yes	244/104 (70%/30%)	176/47 (79%/21%)	420/151 (74%/26%)	0.020
Asthma, no/yes	318/29 (92%/8%)	221/5 (98%/2%)	539/34 (94%/6%)	0.002
ECP434(G>C) GG/GC/CC	190/146/13 (54%/42%/4%)	147/73/5 (65%/32%/2%)	337/219/18 (59%/38%/3%)	0.030
ECP562(G>C) GG/GC/CC	231/110/7 (66%/32%/2%)	169/53/3 (75%/24%/1%)	400/163/10 (70%/28%/2%)	Ns
ECP c.-38(A>C) AA/AC/CC	77/182/89 (22%/52%/26%)	39/115/71 (17%/51%/32%)	116/297/160 (20%/52%/28%)	Ns

### Respiratory symptoms and asthma

The subjects underwent a structured interview, which included detailed information on respiratory symptoms and asthma. The symptoms included in this analysis had all occurred during the preceding 12 months and were: wheeze; wheeze in combination with breathlessness; wheeze when not having a cold, nocturnal chest tightness; attacks of breathlessness following activity, at rest or at night time; and nocturnal cough. "Asthma" was defined as having physician-diagnosed asthma and having had asthma-related symptoms or attacks of asthma in the preceding 12 months [11].

### Allergy testing

Total and specific serum IgE was determined using the Pharmacia CAP System (Phadia AB, Uppsala, Sweden). Specific IgE was measured against *Dermatophagoides pteronyssinus*, timothy grass, cat and *Cladosporium herbarum*. Detection of specific IgE (≥ 0.35 kU/L) was used as the definition of sensitisation. Atopy was defined as being sensitised to any of the above allergens. In this analysis allergic asthma was defined as having asthma in combination with atopy, while non-allergic asthma was defined as having asthma but not having atopy [12].

### Smoking history

Information on smoking history was retrieved from the main questionnaire [13]. Those who answered "yes" to the lead question ("Have you ever smoked for as long as a year?") were classified in *ex-smokers *and *current smokers *based on a negative/positive answer to the question regarding the current consumption ("Do you now smoke, as for one month ago?").

### Lung function

Forced expiratory volume in one second (FEV_1_) was measured. Up to five technically acceptable blows were determined. The ATS recommendations were followed [14]. The predicted values for FEV_1 _were calculated on the basis of the European Coal and Steel Union reference values [15].

### DNA extraction

Genomic DNA was extracted from EDTA-containing whole blood samples collected from 465 individuals of the Swedish population and 292 individuals of the Estonian population. The protocol from the manufacturer of the automated BioRobot M48 System (Genovision, Qiagen GmbH, Hilden, Germany), designed for the MagAttract^® ^DNA Blood Mini M48 Kit (Qiagen), based on magnetic-bead technology was used. DNA from 200 μL of blood was eluted in an equal volume of elution buffer. A limited number of reference samples used for DNA sequencing were extracted by a method previously described by Kawasaki with minor modifications [8,16].

### Genotyping

Three single nucleotide polymorphisms (SNPs) in the ECP gene, ECP c.-38(A>C) rs2233859, ECP434(G>C) rs2073342, and ECP562(G>C) rs2233860 were genotyped by the TaqMan 5' nuclease allelic discrimination assay (Applied Biosystems, Foster City, CA, USA), as previously described [17,18]. Primers and probes (Table 2) were designed according to the guidelines of the manufacturer. Each PCR (polymerase chain reaction) contained template DNA, TaqMan^® ^Universal PCR Master Mix or TaqMan^® ^Genotyping Master Mix (Applied Biosystems), minor grove binding (MGB) probes (200 nmol/L and primers (700 or 900 nmol/L) (Applied Biosystems) and non-acetylated bovine serum (100 or 200 μg/mL) (Sigma-Aldrich, St. Louis, MO, USA). The PCR amplification was run according to the standard protocol consisting of 50°C for 2 min, 95°C for 10 min, and 40 cycles of 95°C for 15 s, followed by 60°C for 1 min. The genotype was determined by use of the application Allelic Discrimination of the ABI PRISM 7000 SDS software. Each run included three non-template controls and two positive controls of each genotype previously analysed by the use of PCR and restriction fragment length polymorphism analysis (RFLP) in case of ECP434(G>C) or sequencing as previously described in case of ECP434(G>C), ECP562(G>C) and ECP c.-38(A>C) [6,8]. Samples genotyped as ECP562CC by the TaqMan^® ^method described above (Method 1) were re-genotyped after amplification of a 870 base pair (bp) fragment of the ECP gene by PCR, followed by the TaqMan 5'nuclease Allelic Discrimination assay (Method 2, Table 2).

**Table 2:** ECP primers and probes derived from the gene sequence of GenBank accession number X16545

Polymorphism	Primer	Probe^a^
ECP c.-38(A>C)	^b^FP: 5'-ATA GTT TTC ACC CAG AGT CCA-3'	
(rs2233859)	^b^RP: 5'-TGC CCG CAT TGC AAT GGT GCA TCG A-3'	
	^c^FP: 5'-CAC CCA GAG TCC AGA TCC CAC CG-3'	
	^c^RP: 5'-GCA TTG CAA TGG TGC ATC GAG-3'	
ECP c.-38(A>C)	FP: 5'-GCC TGT GGG TTG AGA CAC TAT AGA-3'	C: 5'-VIC-AGT AAC TAC TCC CCg ATC C-3'
	RP: 5'-ATT TGG GAA GTG AAC AGT TTT GG-3'	A: 5'-6-FAM-AGT AAC TAC TCC CCt ATC C-3'
ECP434(G>C)	FP: 5'-GAG TAG ATT CCG GGT GCC TTT ACT-3'	G: 5'-VIC-AAA CTG CAg GTA TGC AGA-3'
(rs2073342)	RP: 5'-CGT GGA GAA TCC CGT GGA T-3'	C: 5'-6-FAM-AAA CTG CAc GTA TGC AG-3'
ECP562(G>C)^d^	FP: 5'-GGT TCC AGT TCA CCT GGA TAC C-3'	G: 5'-VIC-TCA GCA gTC CTC ATC-3'
(rs2233860)	RP: 5'-GGT ATG GAG ACT GAT GAG GAC AGT-3'	C: 5'-6-FAM-TCA GCA cTC CTC ATC-3'
ECP562(G>C)^e^	^f^FP: 5'-AAA TGC GAC CCC AGA GTG GCA-3'	
	^f^RP: 5'-AGG GGG AGA GAT TCA TGA TAA ACC C-3'	
	FP: 5'-GGT TCC AGT TCA CCT GGA TAC C-3'	G: 5'-VIC-TCA GCA gTC CTC ATC-3'
	RP: 5'-TGA TTG AGG AGC TTG GCA GAT-3'	C: 5'-6-FAM-TCA GCA cTC CTC ATC-3'

FP: forward primer; RP: reverse primer, a: Polymorphic bases are indicated by lower cases, b: PCR primers, c: sequencing primers, d: Method 1, e: Method 2, f: amplification of a fragment of the ECP gene

### DNA Sequencing of the ECP c.-38 (A>C) polymorphism

A 710 bp fragment of the ECP gene was amplified in a 50 μL PCR reaction containing, template DNA, Platinum^® ^*Taq *DNA Polymerase (1.0 unit), PCR buffer, 2'-deoxynucleoside 5'-triphosphate (dNTP mixture) (0.05 mmol/L), MgCl_2 _(1.5 mmol/L) (Invitrogen, Carlsbad, CA, USA), non-acetylated bovine serum (160 μg/mL) (Sigma-Aldrich) and primers (0.2 μmol/L) (Thermo Electron GmbH, Ulm, Germany) (Table 2). The PCR reactions were run on a Thermo Cycler SDS instrument (MJ Research, Inc., Waltham, MA, USA) with the following PCR profile: 94°C for 30 s, followed by 30 cycles of 94°C for 30 s, 62°C for 30 s, and 72°C for 1 min, and terminated by an additional extension for 5 min at 72°C.

The PCR products were analysed by agarose gel (1%) electrophoresis, and purified by the QIAquick^® ^PCR Purification Kit (Qiagen). The sequencing PCR was set up with the GenomeLab™ Dye Terminator Cycle Sequencing (DTCS) Quick Start Kit (Beckman Coulter, Inc., Fullerton, CA, USA), and subjected to Thermal Cycling in the SDS instrument according to the protocol of the manufacturer. The PCR products were ethanol precipitated, dissolved in Sample Loading Solution (SLS), and loaded into the CEQ™ 8000 DNA analysis system. Subsequently, the nucleic acid sequences were analysed using the software Vector NTI Advance™ 9.0 Software package (InforMax, North Bethesda, MD, USA).

### Statistics

In the comparison of mean and/or median levels of more than two groups we used both parametric ANOVA and the non-parametric Kruskal-Wallis test. In the calculation of differences between two groups either t-test or Mann Whitney's non-parametric test were used. In the calculations of differences in proportions the χ^2^-test or Fisher's exact test were used. In our calculations we made no assumptions as to the inheritance patterns of the alleles. Logistic and multiple regression analyses were performed to study interactions and adjust for confounders. In the models gender, ethnicity, smoking habits were included as categorical data together with the three genotypes. In this way association to atopy, asthma-like symptoms and smoking habits were examined as dependent variables in forward and backward analyses. In our calculations only the randomly selected population of subjects was included unless otherwise stated. In all calculations the statistical softwares Statistica for Windows v. 9 (Tulsa, OK, USA) or Medcalc v. 9 (Mariakerk, Belgium) were used.

## Results

### Demographics

Some of the differences and similarities between the two cohorts from Sweden and Estonia are given in table 1. This shows that more women and smokers were included in the Estonian cohort. More subjects with atopy were included in the Swedish cohort. The overall prevalence of atopy was 27%, with 30% in the Swedish cohort and 21% in the Estonian cohort, p = 0.02. A difference was seen in the prevalence of asthma since 2% of the Estonian cohort was diagnosed with asthma as compared to 8% in the Swedish cohort (p = 0.002). We found significant differences in genotype and allele frequencies between the Swedish and Estonian cohorts with the ECP434GG genotype and ECP434 G-allele more common among Estonians (p = 0.030 and p = 0.017, respectively), but with no differences in ECP562(G>C) or ECP c.-38(A>C) genotype frequencies.

Atopy and smoking habits were similar between genders, whereas the prevalence of asthma was slightly higher among women (p = 0.05) (Table 3). The prevalences of the ECP434 and ECP562 G-alleles were significantly higher among women (p = 0.004 and 0.018, respectively), whereas no gender differences were seen for the ECP c.-38(A>C) gene polymorphism.

**Table 3:** Differences and similarities between males and females in the randomly collected cohort

	Male	Female	All	p-value (χ^2^-test)
Never-smokers/Ex-smokers/Smokers	121/79/76 (44%/29%/28%)	157/75/66 (53%/25%/22%)	278/154/142 (47%/27%/26%)	Ns
Atopy, no/yes	193/82 (70%/30%)	227/69 (77%/23%)	420/151 (74%/26%)	Ns
Asthma, no/yes	267/11 (96%/4%)	272/23 (92%/8%)	539/34 (94%/6%)	0.05
ECP434(G>C) GG/GC/CC	143/127/9 (51%/46%/3%)	194/92/9 (65%/32%/3%)	337/219/18 (59%/38%/3%)	0.002
ECP562(G>C) GG/GC/CC	175/101/2 (63%/36%/1%)	225/65/8 (76%/21%/3%)	400/163/10 (70%/28%/2%)	0.0002
ECP c.-38(A>C) AA/AC/CC	48/152/79 (17%/54%/28%)	68/145/81 (23%/49%/28%)	116/297/81 (20%/52%/28%)	Ns

### Relationships of ECP genotypes to smoking and lung functions

Overall current smokers had a higher prevalence of the ECP434 and ECP562 G-alleles than the non-smoking cohort (p = 0.007 and 0.018, respectively). When separated into never smokers, ex-smokers and current smokers, ex-smokers had the lowest prevalence of the ECP434 or ECP562 G-alleles (p = 0.001 and p = 0.0001, respectively), with no differences between never smokers and current smokers (Table 4). Smoking women had a significantly higher prevalence of the ECP434 and ECP562 G-alleles than smoking men (91% vs 77% and 94% vs 84%, p = 0.002 and p = 0.02, respectively).

**Table 4:** The prevalence of the three ECP genotypes in the never-smoking, ex-smoking and smoking random cohorts of Sweden and Estonia

	Never-smokers	Ex-smokers	Smokers	p-value (χ^2^-test)
ECP434(G>C) GG/GC/CC	170/97/9 (62%/35%/3%)	65/80/7 (43%/53%/5%)	96/42/2 (69%/30%/1%)	0.0001
ECP562(G>C) GG/GC/CC	197/73/6 (71%/26%/2%)	88/60/3 (58%/40%/2%)	109/30/1 (78%/21%/1%)	0.0048
ECP c.-38(A>C) AA/AC/CC	59/142/75 (21%/51%/27%)	22/85/44 (15%/56%/29%)	33/66/41 (24%/47%/29)	Ns

**Figure 1:**
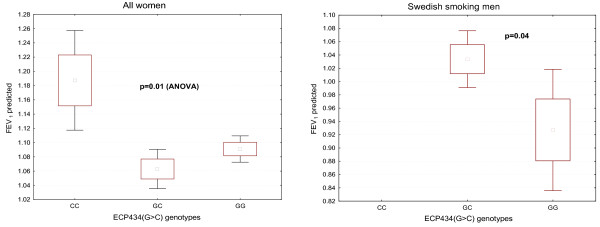
**Lung function (FEV_1 _predicted) in relation to ECP434(G>C) genotypes**. The relation is shown for two cohorts: All women (left panel) and Swedish smoking men (right panel). The statistics is shown in the figure.

Lung function as measured by FEV_1 _predicted was similar among the ECP genotypes. However, among women a significant relationship was found with higher FEV_1 _predicted in those carrying the ECP434CC genotype as compared to ECP434GC (p = 0.01) and as compared to ECP434GG (p = 0.03) (Figure 1). This relationship was only seen among non-smoking women (p = 0.006). A similar relationship was seen to the ECP562(G>C) genotype, but not to the ECP c.-38(A>C) genotype.

### Relationships of ECP genotypes to atopy and asthma

Among males, but not among women, atopy was associated to the ECP c.-38(A>C) genotypes with significantly higher frequency of the CC-genotype (p = 0.007). In a logistic regression analysis the ECP c.-38CC genotype was independently associated with an increased risk of atopy in the randomly selected group (OR 1.9, CI 1.2-3.1, p = 0.007) when adjusted for gender, ethnicity and smoking habits. In order to evaluate any associations to asthma and asthma-like symptoms the whole cohort of subjects was included in the calculations, since the number of subjects with asthma in the randomly selected group was very limited. We did not find any relationships between asthma and genotypes in the entire cohort. By logistic regression analysis with adjustment for gender, ethnicity and smoking habits we confirmed the apparent absence of any associations. However, in the non-smoking cohort the ECP434GG genotype was significantly more common in subjects with non-allergic asthma (p = 0.016). This is illustrated in table 5 by the non-smoking Swedish cohort in which 83% of the non-allergic asthmatics carried the ECP434GG genotype as compared to 48% of the subjects with allergic asthma (p = 0.01). Non-allergic asthma was significantly more prevalent among women (women 46% vs men 16%) and allergic asthma more prevalent among men (women 54% vs men 84%) (p = 0.002). Subjects with non-allergic asthma, in contrast to subjects with allergic asthma, more frequently carried the ECP434GG genotype as compared to non-asthmatics (83% vs 51%, p = 0.006).

**Table 5:** The associations of the ECP434(G>C) gene polymorphism to symptoms and signs of asthma and allergy in the non-smoking Swedish population (random and symptomatic sample)

		434GG	434GC	434CC	p-value (χ^2^-test)
Asthma	Yes No	46 (58%) 155 (51%)	30 (38%) 129 (43%)	3 (4%) 15 (5%)	Ns
Allergic Asthma	Yes No	27 (48%) 19 (83%)	27 (48%) 3 (13%)	2 (4%) 1 (4%)	p = 0.01
Asthma-like symptoms to house dust	Yes No	19 (73%) 183(54%)	5 (20%) 157(42%)	2 (7%) 16 (4%)	p < 0.05
Asthma-like symptoms to pollen	Yes No	38 (63%) 163 (51%)	16 (27%) 146 (45%)	6 (10%) 12 (4%)	p = 0.007

A higher frequency of the ECP434GG genotype was found among the non-smoking subjects who experienced asthma-like symptoms at exposure to house dust (p = 0.04) or to pollen (p = 0.03). Associations to these symptoms (p < 0.05 and p = 0.007, respectively) are illustrated in table 5 for the non-smoking Swedish population. Any associations were not seen to the ECP562(G>C) and ECP c.-38(A>C) genotypes.

## Discussion

In previous studies we showed a relation between the ECP gene polymorphism 434(G>C) and the expression of allergic symptoms [8]. The aim of this study was to confirm and extend this observation in a larger cohort. Three major confounders were identified that interfered with the interpretation of our data. One was the difference in genotypes between the Estonian and Swedish cohorts, the other a gender difference and third and most unexpectedly a close relationship between smoking habits and the ECP434(G>C) genotypes. Thus, in the Estonian cohort the C-allele was under represented as compared to the Swedish population and also as compared to previous population studies in Sweden. Likewise was the C-allele under represented among women. A similar gender relationship was also seen for the ECP562(G>C) polymorphism, which is expected based on the previous findings of very close relationships between these two genotypes with an r^2 ^of 0.64 (p < 0.001) [6]. However, the ECP c.-38(A>C) polymorphism did not show such differences. Overall the genotype frequencies of the ECP562(G>C) and ECP c.-38(A>C) were consistent with Hardy-Weinberg equilibrium, whereas the ECP434(G>C) genotype frequencies were consistent with Hardy-Weinberg equilibrium in the Estonian cohort, but not in the Swedish cohort (χ^2 ^= 6.2, p = 0.01).

The findings of strong associations between smoking habits and the ECP434(G>C) and 562(G>C) genotypes were unexpected and not easily explained. It is important to emphasize that the relations to smoking habits were seen in both ethnic groups, which makes the association more likely. Genetic relations to smoking habits, however, have been documented extensively by others and include relations to nicotine receptor polymorphisms, the dopamine and serotonin systems [19,20]. In a recent genome wide association study candidate genes for successful smoke cessation was performed and showed candidate genes spread over the entire genome, but within certain clusters [21]. Several candidate genes were found on chromosome 14, which is the location of the ECP gene. Three of these are located up streams the ECP gene with distances from the ECP gene between 108 and 143 kbp. One of these is the RNASE6 gene. However, a HapMap survey of a 500 kbp region around the ECP gene revealed no linkages of interest apart from the known linkage to the EPX/EDN gene (to be published Jönsson et al.) and to the ECP pseudogene and several intergene SNPs.

In an unpublished study we previously found a relationship between lung function and the ECP434(G>C) genotype in women, since women who carried the GG genotype had significantly reduced lung function as measured by FEV_1_. Based on these findings we hypothesized that ECP is involved in the impairment of lung function and that those who express the non-cytotoxic variant of ECP are protected from the consequences of the detrimental effects of invading eosinophils in diseases such as seen in smokers, asthma and COPD. Our findings in the present study also showed a relationship between lung function and the genotypes of ECP434(G>C) and ECP562(G>C), since women who carried the CC genotypes had significantly higher FEV_1 _predicted. This association was found irrespective of smoking habits, but was significant only among non-smoking women. The numbers, however, of ever smoking women carrying the CC genotypes were very few, which precluded any further calculations. Thus, our previous hypothesis had some support from these findings. The need of further studies to validate our findings is obvious.

We confirmed previous results of a relationship between expression of allergic symptoms and the ECP434(G>C) genotypes, [8] since self reported complaints of allergic symptoms in contact with pollen and house dust were more frequent among those who carried the ECP434GG genotype. Also none of the subjects in the randomly selected group of subjects who reported symptoms of asthma carried the ECP434CC genotype. One paradox, however, was that non-allergic asthma was closely related to this genotype as well, in contrast to allergic asthma, since non-allergic asthma primarily was seen in women who carried the ECP434GG genotype. This is obviously contradicting our previous findings in which we found a higher prevalence of the ECP434GG genotype among allergic asthmatics [8]. In the previous study the distinctions were based on doctor's diagnosis of asthma, whereas the distinction in this survey was based on questionnaires and the retrospective diagnosis of the investigators. Moreover, our study included both randomly selected subjects with asthma and an additional cohort of subjects with a confirmed history of asthma, which may have biased our results slightly. Whether this explains the seeming paradox is unknown at present, but another study on Norwegian and Dutch subjects with asthma found a relation of this genotype to non-allergic asthma [7]. Further studies are needed to settle this matter. A confounder in the present study was the high prevalence of the ECP434GG genotype in women, which paralleled the predominance of non-allergic asthma among women.

In the above mentioned study on Norwegian and Dutch subjects relationships between the ECP c.-38(A>C) genotypes and signs of asthma and serum IgE levels were found in addition to a relationship to serum ECP levels [7]. We also found associations to atopy, but our findings were opposite since atopy was more prevalent in subjects carrying the ECP c.-38CC genotype, whereas the A-allele seemed more prevalent in the Norwegian-Dutch study. Our findings showed by logistic regression analysis that this association was independent when adjusted for the major confounders; gender, ethnicity and smoking habits. However, in separate evaluations of sub groups the association to atopy were only seen in the Swedish cohort and most obvious among non-smokers and males. In contrast to the two ECP gene polymorphisms 434(G>C) and c.-38(A>C) we found very few associations to the third gene polymorphism i.e. ECP562(G>C). This confirms our previous study and shows that the cellular content of ECP is not a major determinant of disease development, [6] whereas the quality in terms of cytotoxic activity of the protein seems more important. Theoretically, the combination of the genotypes should give us stronger disease associations. However, this was not the case (results not shown), since the strongest relationships were found with single genotype polymorphisms and not haplotypes of the three genotypes. In this report we did not correct statistically for multiple comparisons, which may limit our conclusions. However, the fact that we saw relationships to several allergic manifestations would support our notions of the impact of the ECP genotypes.

## Conclusion

We conclude from this study that the three common ECP genotypes are related to several signs and symptoms of allergy and asthma, but that confounders such as gender, ethnicity and smoking habits have to be considered in the evaluations. The most conspicuous results were the strong relationships to smoking habits and lung function of the ECP434(G>C) genotypes, which suggests that the activity of ECP may be of importance for the development of irreversible damage to the lung in smokers and pulmonary diseases. Indeed ECP is highly cytotoxic and able to destroy lung epithelial cells, [1,22] but may also take part in the remodelling of the lungs seen in smokers [23].

## Competing interests

The authors declare that they have no competing interests.

## Authors' contributions

UBJ analysed the samples and prepared the preliminary manuscript. LDH was instrumental in the establishment of the TaqMan assays. RJ was responsible for the collection and clinical work up of the Estonian cohort. CJ was responsible for the collection and clinical work up of the Swedish cohort. PV supervised the study and prepared the final manuscript. All authors have read and approved the results and conclusions of the manuscript.

## Pre-publication history

The pre-publication history for this paper can be accessed here:

http://www.biomedcentral.com/1471-2466/10/36/prepub
